# Insomnia among patients with chronic pain: A retrospective study

**DOI:** 10.1097/MD.0000000000039113

**Published:** 2024-08-09

**Authors:** Masataka Ueda, Tomoko Tetsunaga, Tomonori Tetsunaga, Keiichiro Nishida, Ryo Takatori, Hisakazu Shitozawa, Koji Uotani, Kennsuke Shinohara, Yoshiaki Oda, Toshifumi Ozaki

**Affiliations:** a Department of Orthopaedic Surgery, Science of Functional Recovery and Reconstruction, Okayama University Graduate School of Medicine, Dentistry and Pharmaceutical Sciences, Okayama, Japan; b Department of Orthopaedic Surgery, Okayama University; c Department of Musculoskeletal Health Promotion, Faculty of Medicine, Dentistry and Pharmaceutical Sciences, Okayama University, Okayama, Japan; d Division of Chronic Pain Medicine and Division of Comprehensive Rheumatology, Locomotive Pain Center, Okayama University Hospital; e Department of Sports Medicine, Faculty of Medicine, Dentistry and Pharmaceutical Sciences, Okayama University; f Department of Orthopaedic Surgery, Science of Functional Recovery and Reconstruction, Faculty of Medicine, Dentistry and Pharmaceutical Sciences, Okayama University.

**Keywords:** AIS, cognitive-behavioral therapy, EQ5D, HADS, insomnia, pain-liaison outpatient clinic, sleep disorders

## Abstract

Insomnia can coexist with chronic pain and is a major cause of rapidly increasing medical expenses. However, insomnia has not been fully evaluated in patients with chronic pain. This retrospective study aimed to identify the risk factors for insomnia in patients with chronic non-cancer pain. A total of 301 patients with chronic non-cancer pain were enrolled. Patients with the Athens insomnia scale scores ≥ 6 and < 6 were classified into insomnia (+) and insomnia (−) groups, respectively. All patients completed self-report questionnaires as part of their chronic pain treatment approach. Univariate and multivariate analyses were performed to predict insomnia. We found that 219 of 301 (72.8%) patients met the AIS criteria for insomnia. Significant differences were depicted between patients with and without insomnia in terms of body mass index, numeric rating scale, pain catastrophizing scale, hospital anxiety, and depression scale (HADS), pain disability assessment scale, EuroQol 5 dimension (EQ5D), and pain self-efficacy questionnaire. Multiple regression analysis identified the numeric rating scale, HADS, and EQ5D scores as factors related to insomnia in patients with chronic non-cancer pain. Anxiety, depression, and disability were associated with a greater tendency toward insomnia. HADS and EQ5D scores are useful screening tools for preventing insomnia in patients with chronic non-cancer pain.

## 1. Introduction

**Figure 1.**
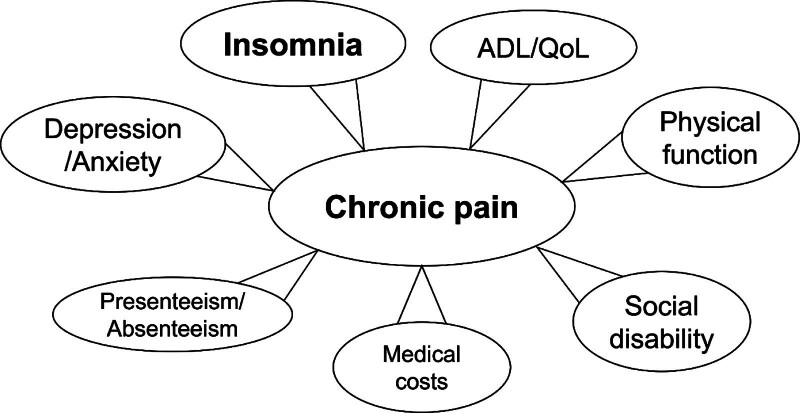
Multifaceted impact of chronic pain.

Chronic pain and insomnia are prevalent health issues in modern society. Chronic pain is defined as pain persisting for 3 months or longer, exceeding the expected treatment duration.^[1]^ The impact of chronic pain is multifaceted, as illustrated in Figure 1. It affects activities of daily living (ADL) and quality of life (QoL),^[2]^ leading to depression, anxiety,^[3]^ increased medical costs,^[4]^ presenteeism, absenteeism,^[5,6]^ and insomnia.^[3]^ Insomnia, defined as the inability to obtain sufficient quality and quantity of sleep, adversely affects health and QoL. Chronic pain and insomnia share a close relationship, mutually influencing each other, with numerous reports exploring this connection.^[7–12]^ Insomnia affects 10% to 15% of the general population,^[11]^ and research indicates that clinical insomnia is prevalent in over half of the patients with chronic pain, surpassing general population rates.^[8]^ Many individuals with chronic pain struggle with falling asleep or experience sleep disruptions due to pain and insomnia. Notably, insomnia can amplify the perception of pain, intensifying its impact.^[12]^ Such interactions can adversely affect a patient’s QoL and daily functioning and can lead to psychological distress.^[13]^ Furthermore, sleep disturbance may compromise pain control, contributing to chronic pain.^[7]^ Sleep disorders can heighten pain sensitivity, triggering spontaneous pain, escalating the risk of developing chronic pain, and worsening existing pain.^[9]^ However, the factors influencing insomnia in patients with chronic pain remain unclear.

This study aimed to investigate the factors associated with insomnia in individuals with intractable chronic pain. We posit that the factors influencing insomnia in patients with chronic non-cancer pain, identified in this study, could serve as new therapeutic targets.

## 2. Methods

### 2.1. Study participants

**Figure 2.**
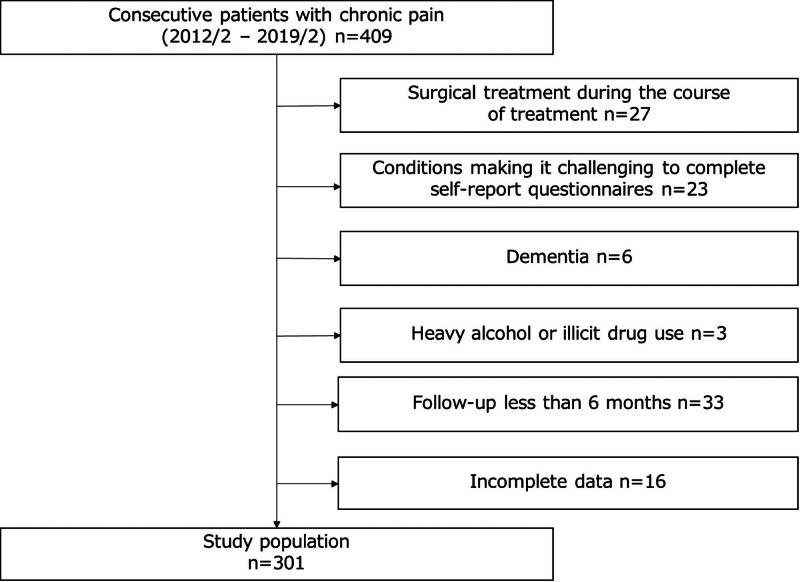
Flowchart showing study population and patient recruitment.

Ethical approval was obtained from our institution’s Institutional Review Board, and we used the Strengthening the Reporting of Observational studies in Epidemiology reporting guidelines for cohort studies. All patients provided written informed consent for self-report questionnaires as part of their chronic pain treatment approach. This retrospective study enrolled 301 patients (109 men and 192 women) with chronic non-cancer pain admitted to our hospital between February 2014 and February 2019. Inclusion criteria involved patients with persistent non-cancer pain for at least 3 months and their agreement to complete questionnaires. Exclusion criteria included patients with dementia, heavy alcohol or illicit drug use, those who received surgical treatment during the course of treatment, those with conditions making it challenging to complete self-report questionnaires, those who were lost to follow-up in <6 months, and those with incomplete data (Fig. 2).

Patients were divided into 2 groups based on their Athens insomnia scale (AIS) scores.^[14]^ The AIS, a straightforward diagnostic tool for insomnia, efficiently assesses a patient’s sleep status. It consists of 8 items, facilitating the determination of insomnia presence. The 8 multiple-choice items in the AIS can be easily adjusted to modify references to sleep induction, awakening during the night, final awakening, total sleep duration, sleep quality, well-being during the day, functioning capacity during the day, and sleepiness during the day. Scores for each item ranged from 0 (no problem) to 3 (very serious problem), with a total score range of 0 to 24. The total score cutoff for identifying pathological insomnia in AIS is 6 points.^[14]^ Additionally, demographic and clinical background data were collected from the patients.

### 2.2. Pain assessment

The numeric rating scale (NRS) is a widely used, valid, and reliable tool for measuring the intensity of chronic non-cancer pain. NRS scores range from 0 to 10, with 0 representing no pain and 10 representing the worst pain imaginable.

### 2.3. Physical disability assessment

The items in the pain disability assessment scale (PDAS) were designed to evaluate the negative effects of pain on various pain interference domains.^[15]^ The PDAS consists of 20 items rated on a 4-point Likert scale, with scores ranging from 0 to 60 points. Clinicians seeking a multidimensional measure of pain use the PDAS to examine patients’ daily routines.

### 2.4. Anxiety and depression assessment

The hospital anxiety and depression scale (HADS) is employed for assessing anxiety and depression.^[16]^ Patients with physical illnesses can be easily evaluated for anxiety and depression using the HADS. This 14-item scale consists of 7 items assessing anxiety and 7 items assessing depression. Each item is rated from 0 to 3 on a 4-point Likert scale. Overall scores for anxiety or depression range from 0 to 21, with higher scores indicating greater symptom severity.

### 2.5. Pain catastrophizing assessment

The pain catastrophizing scale (PCS) was used to assess self-reported pain catastrophizing owing to chronic pain.^[17]^ The PCS, a broad measure of pain catastrophizing, is composed of 13 items rated from 0 (never) to 4 (always) on a 5-point Likert scale. The maximum score for the PCS is 52, with higher scores indicating greater pain catastrophizing levels. High levels of catastrophizing were defined as scores > 24. The items on the PCS were divided into the following 3 subscales: rumination, helplessness, and magnification. Rumination (items 8–11) “refers to the fact that the patient cannot get the idea of pain out of his/her head and cannot stop thinking about the pain.” Helplessness (items 1–5 and 12) “refers to the estimation that the person has not been able to do anything to influence the pain.” Magnification (items 6, 7, and 13) “refers to the exaggeration of the threatening properties of the painful stimulus.”

### 2.6. Health-related QoL assessment

Health-related quality of life (HRQoL) reflects overall persistent or recurrent pain. This is measured using the EuroQol 5 dimension (EQ5D), which has been validated in patients with persistent pain.^[18]^ The EQ-5D defines the individual’s health status by a single summary index ranging from 0 to 1, where 0 corresponds to death, and 1 corresponds to full health.^[18,19]^ The questionnaire is reliable and validated in Swedish.^[19,20]^

### 2.7. Self-efficacy assessment

The pain self-efficacy questionnaire (PSEQ) is a 10-item questionnaire developed to assess the confidence of individuals with ongoing pain in performing activities in pain. The PSEQ applies to all presentations of persistent pain. It inquiries into a level of self-efficacy regarding a range of functions, including household chores, socialization, work, and coping with pain without medication. The raw score ranges from 0 to 60, with high scores indicating greater levels of confidence in dealing with pain. High scores are strongly associated with clinically significant functional levels and provide a useful gauge for evaluating outcomes in patients with chronic pain.^[21]^

### 2.8. Statistical analysis

Patients with AIS scores ≥ 6 and < 6 were classified into insomnia (+) and insomnia (−) groups, respectively. Univariate analyses were conducted between the groups to compare age, sex, body mass index (BMI), NRS, PDAS, HADS, PCS, EQ5D, PSEQ, and AIS scores. Sex was compared using chi-squared test, and other normally distributed variables were compared using Student *t* test. The chi-squared analysis was performed for categorical variables. Statistical significance was set at *P < *.05. The factors predicting insomnia were identified using multivariate analysis (multiple regression). Potential predictive variables were included in the multivariate model if values of *P < *.05 were obtained on univariate analysis. A multiple regression model and 95% confidence intervals (CIs) were used to identify the risk factors for insomnia. Statistical analyses were conducted using SPSS software version 25.0 for Windows (IBM, Tokyo, Japan).

## 3. Results

### 3.1. Patients’ background

Table 1 shows the characteristics of the patients involved in this study. The mean age of the participants at the time of examination was 61.7 ± 13.1 years (range, 40–92 years), with a mean duration of pain from onset of symptoms of 47.9 ± 63.4 months (range, 3 months–24 years). Table 2 shows the distribution of pain locations among the participants. Among all patients, 219 (72.8%) met the criteria for insomnia based on their AIS scores.

**Table 1:** Patients’ characteristics.

Variables	n = 301
Age (yr)	61.7 ± 13.1
Sex (men/women)	109/192
BMI (kg/m^2^)	22.6 ± 4.0
NRS	6.0 ± 2.2
PCS	35.3 ± 11.0
HADS anxiety	7.9 ± 4.4
HADS depression	8.9 ± 4.9
PDAS	26.4 ± 13.7
EQ5D	0.54 ± 0.17
PSEQ	25.1 ± 1.5
AIS	8.9 ± 4.9

AIS = Athens insomnia scale, BMI = body mass index, EQ5D = EuroQol 5 dimension, HADS = hospital anxiety and depression scale, NRS = numeric rating scale, PCS = pain catastrophizing scale, PDAS = pain disability assessment scale, PSEQ = pain self-efficacy questionnaire. Parameter values are expressed as mean ± standard deviation or number.

**Table 2:** Pain location.

Location	n = 301
Entire body	30
Face	32
Upper extremity	40
Shoulder	15
Neck	17
Lumbar	46
Trunk	24
Abdomen	8
Buttock	17
Lower extremity	44
Hip	12
Knee	3
Foot	13

### 3.2. Univariate analyses based on the presence of insomnia

The patients were divided into 2 groups based on the presence of insomnia. The insomnia (−) group consisted of 82 patients (30 men and 52 women). The insomnia (+) group comprised 219 patients (79 men and 140 women). Table 3 shows the results of a univariate analysis assessing the presence of insomnia. Age or sex showed no significant differences between the groups (*P* = .0818 and.9344, respectively). The NRS scores were significantly higher in the insomnia (+) group compared to the insomnia (−) group (*P < *.001). The mean BMI in the insomnia (+) group was lower than that in the insomnia (−) group (*P = *.0296). Other questionnaire scores were significantly different between the 2 groups (all *P < *.001). As shown in Table 3, PCS, HADS, PDAS, and PSEQ scores (all *P < *.001) were significantly higher in the insomnia (+) group compared to the insomnia (−) group. These results showed that patients with insomnia experienced more severe pain, pain catastrophizing, anxiety, depression, pain disability, and pain self-efficacy than those without insomnia.

**Table 3:** Comparison of pain-related parameters with and without insomnia.

Variables	Insomnia		*P* value
	(−) (n* *= 82)	(+) (n = 219)	
AIS	3.4 ± 1.3	11.0 ± 4.1	<.001*
Age (yr)	63.8 ± 13.1	60.9 ± 13.1	.0818
Sex (men/women)	30/52	79/140	.9344
BMI (kg/m^2^)	23.5 ± 4.5	22.3 ± 3.7	.0296*
NRS	4.8 ± 2.2	6.4 ± 2.0	<.001*
PCS	28.8 ± 12.7	37.7 ± 9.1	<.001*
HADS anxiety	4.7 ± 2.8	9.0 ± 4.3	<.001*
HADS depression	5.5 ± 3.6	10.2 ± 4.7	<.001*
PDAS	21.3 ± 13.1	28.2 ± 13.4	<.001*
EQ-5D	0.6 ± 0.2	0.5 ± 0.2	<.001*
PSEQ	1.7 ± 1.3	34.6 ± 14.6	<.001*

Data are expressed as mean ± standard deviation. AIS = Athens insomnia scale. AIS < 6 is defined as Insomnia (−), and AIS ≧ 6 is defined as insomnia (+). BMI = body mass index, EQ5D = EuroQol 5 dimension, HADS = hospital anxiety and depression scale, NRS = numeric rating scale, PCS = pain catastrophizing scale, PDAS = pain disability assessment scale, PSEQ = pain self-efficacy questionnaire. Sex is compared using a Chi-square test and other parameters are compared using Student *t* tests.

* *P* < .05.

### 3.3. Correlation between factors and insomnia

Factors predicting insomnia were identified using multivariate analysis, as illustrated in Table 4, which shows that insomnia was associated with some of these factors. In such cases, we used a simple linear correlation to evaluate the presence of these potential correlations. Multiple regression analysis was performed to investigate the association between insomnia and no insomnia as the response variable, with NRS, HADS (both anxiety and depression), and EQ5D serving as explanatory variables. We found that the AIS scores (*y*) were positively correlated with NRS (*x*_1_), HADS anxiety (*x*_2_), and HADS depression (*x*_3_), and negatively correlated with EQ5D scores (*x*_4_) (Table 4). The resulting prediction formula is expressed as *y* = 5.12 + 0.432* x*_1_ + 0.266* x*_2_ + 0.219* x*_3_ – 5.23* x*_4_. The adjusted coefficient of determination was 0.397, and all *P* values were < .05, indicating that the variables chosen for analysis in this study had good explanatory power.

**Table 4:** Correlation between factors and Athens insomnia scale (AIS).

Variables	Partial regression coefficient	Standard error	95% CI		*P* value
			Lower	Upper	
NRS	0.4323	0.1194	0.1972	0.6674	<.001*
HADS anxiety	0.266	0.076	0.1164	0.4156	<.001*
HADS depression	0.2192	0.0704	0.0806	0.3578	.0020*
EQ5D	−5.2264	1.6689	−8.5113	−1.9415	.0019*
Constant term	5.124	1.5571	2.059	8.1889	.0011*

CI = confidence interval, EQ5D = EuroQol 5 dimension, HADS = hospital anxiety and depression scale, NRS = numeric rating scale.

* *P* < .05.

## 4. Discussion

We investigated the factors contributing to insomnia in patients with chronic non-cancer pain, using AIS scores. Among the 301 patients analyzed, 219 (72.8%) met the insomnia criteria. Multiple regression analyses revealed that elevated NRS, HADS, and EQ5D scores were associated with insomnia in patients with chronic non-cancer pain. These findings suggest that HADS and EQ5D are useful screening tools for guiding the development of treatments for sleep disorders among patients with chronic nonpain.

As most pain conditions involve various pathways, relying on analgesic therapy using a single agent is inadequate for relieving chronic pain. Chronic pain is a multidimensional problem that must be treated using a combination of biological, psychological, and social approaches.^[22]^ Pincus et al^[23]^ examined the transition to chronic pain and reported strong evidence for the role of negative moods, such as distress or depression. Briefly, in addressing chronic pain, combining the following 3 treatment approaches is important: elimination of the cause, including advanced pain interventions; lifestyle changes with physical therapy; and pharmacotherapy.^[24]^ Notably, cognitive-behavioral therapy is widely accepted for use in patients with chronic pain.^[7,25,26]^ Most patients with intractable chronic pain experience pain due to inactivity and have a strong fear of movement.^[27]^ Therefore, multidisciplinary approaches, including patient education and physical therapy, are necessary to treat patients with anxiety and depression. Patients undergoing treatment for intractable chronic pain at the pain-liaison outpatient clinic showed significant improvement in anxiety, depression, and HRQoL after 6 months of treatment.^[27]^

Sleep disorders negatively affect optimism, sociability, and psychosocial functioning.^[28]^ They contribute to increased depressive and anxiety symptoms,^[29]^ increased focus on pain and pain helplessness,^[30]^ and decreased activity.^[31,32]^ Moreover, depression and sleep disorders, which cause functional impairment,^[33]^ can lead to reduced pain thresholds.^[34]^ The current study revealed that patients with sleep disorders had anxiety, depression, and decreased HRQoL.

**Figure 3.**
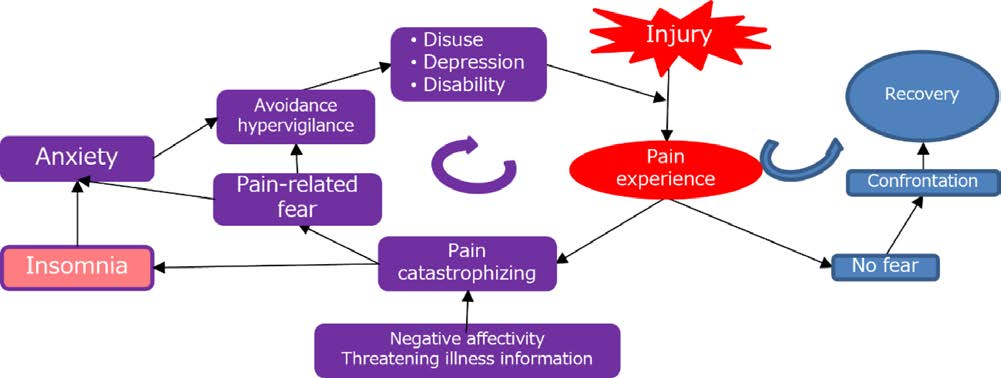
Fear-avoidance model.

Factors beyond pain that contribute to the deterioration of QoL and ADL in patients with chronic pain include comorbidities such as sleep disorders and anxiety.^[35–37]^ Insomnia emerges as the predominant complaint of dissatisfaction with sleep quality and duration, often leading to clinically significant distress or impaired daytime functioning.^[38]^ Approximately 50% of individuals with persistent insomnia develop chronic pain; conversely, approximately half of the patients with chronic pain meet the criteria for persistent insomnia.^[8]^ Elevated pain intensity negatively influences sleep homeostasis by increasing sympathetic nervous system outflow.^[39,40]^ Sleep disorders are associated with an increased mu-opioid receptor-binding potential during evoked pain.^[41]^ Sleep and pain have been reported to interact in a bidirectional manner, with sleep having a stronger causal influence on pain than vice versa.^[9,10]^ The underlying mechanisms are not fully understood; however, sleep dysregulation may heighten pain sensitivity. Sleep disorders can induce systemic inflammation, which is related to elevated pain sensitivity.^[42,43]^ The coexistence of chronic pain and sleep disorders is considered to cause health problems. Both sleep disorders and chronic pain are associated with depression and pain catastrophizing.^[44]^ Considering the fear-avoidance model (Fig. 3), improving insomnia could reduce anxiety (hypersensitivity response to pain) and fear (pessimistic interpretation).^[45,46]^ Therefore, treatment for insomnia is necessary in parallel with the treatment of chronic pain. The effectiveness of cognitive-behavioral therapy in patients with insomnia has been reported in previous studies.^[47,48]^ We believe that this treatment could also be administered to patients with chronic pain and insomnia.

The key pillars in the treatment strategy for insomnia are the elimination of causative factors, sleep hygiene guidance, and medical treatment. When conditions such as sleep apnea syndrome or restless legs syndrome contribute to insomnia, targeted treatments can be implemented. Davis et al^[49]^ reported that interventions that do not directly target sleep may have positive effects on sleep quality and quantity. For instance, stress management and exercise therapy improve sleep in patients with cancerous and noncancerous pain.^[49,50]^ Inoue et al^[51]^ reported that individuals who walk 30 minutes or more a day for 5 or more days a week have lower rates of difficulty falling asleep and waking up in the middle of the night. We believe that this kind of patient education is effective for those with insomnia. However, medical staff should actively introduce a multidisciplinary approach to such patients when needed because patients with chronic pain rarely seek consultation-liaison psychiatry.^[52]^ Insomnia is “a family illness,” and the patient’s family plays a crucial role in its recovery. Therefore, cognitive-behavioral therapy involving patients’ families is recommended.^[27]^

Sleep-promoting medications can reduce pain and improve sleep in patients with chronic pain. Bohra et al^[39]^ reported that antidepressants and opioids have both positive and negative effects on sleep; some anticonvulsants are sleep-sparing and nonsteroidal anti-inflammatory drugs are sleep-neutral. Pharmacological sleep promotion may decrease pain intensity in patients with chronic pain.^[53]^ Andersson et al^[7]^ reported the potential benefits associated with short- to medium-term (2–8 weeks) melatonin and eszopiclone treatment in patients with chronic pain. However, whether these analgesic effects are mediated by improved sleep quality, psychological factors, or other mechanisms remains unclear. Selection of better sleep-promoting medications is important to improve insomnia associated with pain. We considered the following selection criteria important: less dependence, low risk of falling, rapidity of sleep onset, and low carry-over effects. Further research is required to fully understand the effect of sleep-targeting medications on pain control.

### 4.1. Limitations

The current study has some limitations. First, we did not classify the patients based on the nature and location of chronic pain. Because both neuropathic and nociceptive components contribute to chronic pain, these require different pain management strategies owing to their different pathogenesis.^[9]^ Insomnia could be possibly examined in more detail by classifying patients with chronic pain according to the type of pain. Second, this cross-sectional study did not examine the efficacy of sleep-promoting medications in patients with insomnia. Many of the patients who visited our pain-liaison outpatient clinic had multifaceted components in addition to chronic pain; at the time of their first visit to our clinic, most had been to several other hospitals and had taken several internal medications. For a more detailed study, classification according to the number and type of medications might be necessary. However, such classifications can be complex and difficult to execute comprehensively. Third, we did not investigate the effects of pain-liaison outpatient treatment in patients with chronic pain and insomnia. We consider that a multifaceted approach is necessary to alleviate insomnia in patients with chronic pain and insomnia.

Despite these limitations, this study provided new insights into insomnia. Additionally, based on the correlation found between the HADS and EQ5D scores and insomnia, these scores might be beneficial screening tools that can be used at the time of the first examination to detect insomnia. The results of this study could lead to improvements in the treatment of patients with chronic pain.

## 5. Conclusion

Our study revealed that 72.8% of the patients met the diagnostic criteria for insomnia, indicating a high prevalence of insomnia among patients with chronic pain. The findings suggest that HADS and EQ5D may be useful screening tools for insomnia prevention in patients with non-cancer chronic pain.

## Acknowledgments

We would like to thank Editage for the English language editing.

## Author contributions

**Methodology:** Masataka Ueda, Tomonori Tetsunaga.

**Supervision:** Masataka Ueda, Tomonori Tetsunaga, Keiichiro Nishida, Toshifumi Ozaki.

**Validation:** Masataka Ueda, Keiichiro Nishida, Ryo Takatori, Hisakazu Shitozawa, Koji Uotani, Kennsuke Shinohara, Yoshiaki Oda, Toshifumi Ozaki.

**Visualization:** Masataka Ueda.

**Writing – original draft:** Masataka Ueda.

**Writing – review & editing:** Masataka Ueda, Tomoko Tetsunaga, Tomonori Tetsunaga.

**Conceptualization:** Tomoko Tetsunaga, Tomonori Tetsunaga.

**Data curation:** Tomoko Tetsunaga, Tomonori Tetsunaga.

**Formal analysis:** Tomoko Tetsunaga, Tomonori Tetsunaga.

**Funding acquisition:** Tomoko Tetsunaga, Tomonori Tetsunaga.

**Investigation:** Tomoko Tetsunaga, Tomonori Tetsunaga.
